# Expression of concern: Visible light driven photocatalytic oxidation of thiols to disulfides using iron phthalocyanine immobilized on graphene oxide as a catalyst under alkali free conditions

**DOI:** 10.1039/d4ra90152e

**Published:** 2025-01-17

**Authors:** Pawan Kumar, Garima Singh, Deependra Tripathi, Suman L. Jain

**Affiliations:** a Chemical Sciences Division, CSIR-Indian Institute of Petroleum Mohkampur Dehradun-248005 India suman@iip.res.in +91-135-2660202 +91-135-2525788; b Analytical Science Division, CSIR-Indian Institute of Petroleum Mohkampur Dehradun-248005 India

## Abstract

Expression of concern for ‘Visible light driven photocatalytic oxidation of thiols to disulfides using iron phthalocyanine immobilized on graphene oxide as a catalyst under alkali free conditions’ by Pawan Kumar *et al.*, *RSC Adv.*, 2014, **4**, 50331–50337, https://doi.org/10.1039/C4RA10128F.

*RSC Advances* is publishing this expression of concern in order to alert our readers that we are presently unable to confirm the reliability of the conclusions presented in the article due to concerns that insufficient characterization data was provided.

An investigation is underway, and an expression of concern will continue to be associated with this article until a final outcome is reached.

Laura Fisher, Executive Editor, *RSC Advances*

12^th^ December 2024

